# “Balancing within a closed family system”: a grounded theory study of how family life is affected by having a family member with an eating disorder

**DOI:** 10.1186/s40337-022-00669-9

**Published:** 2022-10-10

**Authors:** Jannike Karlstad, Cathrine F. Moe, Ragni Adelsten Stokland, Berit S. Brinchmann

**Affiliations:** 1 Faculty of Nursing and Health Sciences, Nord University, Bodø, Norway; 2 Nordland Hospital Trust, Bodø, Norway

**Keywords:** Anorexia nervosa, Bulimia nervosa, Family functioning, Qualitative research, Grounded theory

## Abstract

**Background:**

This qualitative study explores how having an adult daughter or sister with anorexia nervosa or bulimia nervosa affects the family’s daily life. Previous research has focused on the family’s role in the development of an eating disorder, while more recently the focus has been on the illness’s impact on the family. Caring for an individual with an eating disorder can involve distress, guilt, extra burdens, and unmet needs. By interviewing the family members of adults with eating disorders this study aims to gain insights into how the family members experience the impact of the eating disorder over time.

**Methods:**

A constructivist grounded theory approach was comprised to gather and analyse data to develop a theory on how families experience life with an adult daughter or sister with an eating disorder. Semi-structured individual interviews were conducted with 21 parents and siblings from across Norway.

**Results:**

The participants reported a lack of professional help and the families had to handle the challenges related to the illness themselves, hence *being left to themselves as a family* was identified as the participants’ main concern. To manage this concern the families had to *balance a pattern of care within the closed family system*, and this comprises the core category of the study. Families balanced a pattern of care by *shifting roles*, *adapting meal routines,* and *maintaining openness within the family.*

**Conclusions:**

The findings implicate that families need clarification of roles and responsibilities in relation to the health services in caring for the individual with an eating disorder. More research on taking a family perspective concerning adults with eating disorders is needed.

## Plain English summary

This study explores how having an adult daughter or sister with an eating disorder affects the family’s daily life. Previous research shows that having a family member with an eating disorder can have a great impact on the family life by causing distress, guilt, extra burdens, and unmet needs for the family members. In this study, we interviewed 21 parents and siblings from across Norway. Our study aimed to explore how the family members experienced the impact of the eating disorder over time, we, therefore, wanted to interview families of adults with eating disorders. The participants reported a lack of professional help and they were left to themselves as families having to handle the challenges related to the illness. The families established a system for taking care of the family member with the eating disorder. Both the parents and the siblings assumed the role of caregiver, the whole family adapted their meal routines and the families managed to keep an openness within the family. These findings show that families need clarification of roles and responsibilities in relation to the health services in caring for a family member with an eating disorder.

## Background

The family’s role in the development and maintenance of eating disorders has been researched for decades [1–4]. In more recent years focus has also been on the impact that eating disorders have on the family. In 1978 Minuchin and colleagues identified characteristics of enmeshment, overprotection, rigidity, and avoidance or non-resolution of conflict in families where one member developed anorexia [1]. Familial influence on anorexia and bulimia was reviewed in 1987 by Strober and Humphrey, who found that no single mechanism or pathway of influence was associated with eating disorders, although certain genetically determined personality factors might predispose the individual to greater sensitivity and vulnerability [5]. Polivy and Herman identified similar findings in a review in 2002, by claiming that familial characteristics could be viewed as not causative in themselves but as additional vulnerability factors in the development of eating disorders. In other words, certain personality features and sociocultural pressure were involved in the development of the illness [6].

Whitney and Eisler [7] reviewed family experiences and processes concerning caring for an individual with an eating disorder and found that blaming the family for the development of eating disorders was poorly supported by empirical evidence. However, the families could be stuck in unhelpful interactions and lose sight of their own resources, which might unintentionally contribute to the maintenance of the eating disorder [7]. Further research on treatment for eating disorders supported this view. Eating disorders should be seen as the ‘common enemy’ by family members. Family members need to help detach themselves from behaviours that enable or accommodate the eating disorder, and this has accordingly become an important part of treatment for eating disorders across the age and diagnostic spectrum [8, 9]. Help and support for families to ease their burden in coping with an eating disorder should be prioritized over accusations and blame [10]. In a review on family functioning from 2014, it was found that families of individuals with eating disorders were reported to have worse family functioning than control families, although little evidence has been found for a typical pattern of dysfunction. The authors suggested that the poorer family functioning could be present before the onset of the eating disorder and become worse when the family had to cope with having a member with this illness [11].

Depression, anxiety, and worry are primary emotional reactions in families affected by an eating disorder. The caregiver burden on families affected by an eating disorder can be immense due to carer tasks and negative impact on everyday life and the relationship between the family members [12–16]. The all-consuming demands of the disorder impact the carer’s ability to attend to and maintain relationships and activities outside the family unit. And the lack of understanding about the illness and support provided by both other family members and the health services exacerbates the family’s feelings of being alone [15]. The family system can be characterised as being closed when there is little contact with others outside the family, while the family members are very close and loyal to each other [17]. Families experience a need for professional help and assistance in relation to their own emotional reactions, and parents report difficulties in responding to their own needs and the needs of other family members [12–14, 18–20]. Siblings in families with eating disorders report issues such as insufficient care and negative changes in family life, and the illness takes up considerable time and space [21–23].

Those caring for individuals with eating disorders have reported extensive unmet needs, mainly regarding support and counselling by health services [12, 14, 16]. An association has been found between the duration of the illness and the caregiving experience, as the caregivers must adapt to the illness to a greater degree when it persists [12, 16, 24]. Hopes of recovery and external assistance tended to be higher during the early phases of the illness [12, 25–27]. Individuals with eating disorders are reported to find independence and adult responsibility difficult, hence they often end up living with their primary family longer than their peers [26, 28, 29].

This study is part of a larger project on the experiences and strategies of being a parent or sibling of an adult woman with an eating disorder. The current article is based on findings from two respective sub-studies on parents’ and siblings’ experiences [30, 31]. By interviewing the family members of adults with eating disorders we aim to gain insights into how the participants have experienced the impact of the eating disorder over time. Moreover, as argued above, most of the focus has been on families with children and adolescents with eating disorders, hence more knowledge about the adult group is needed. The aim of this study is to generate a theory about how family life is affected by an eating disorder. How does having a daughter/sister with an eating disorder affect the family?

## Methods

### Design

This qualitative study used a constructivist grounded theory approach to gather and analyse data to develop a theory on how families experience life with a daughter/sister with an eating disorder [32]. A grounded theory approach was chosen because it is considered appropriate when exploring actions and interactions in particular settings when the aim is to explain rather than describe [32, 33]. The avoidance of a pre-formulated hypothesis means that the explanation is generated from the data, that is, the analysis is ‘grounded’ in the data. The simultaneous collecting and analysis of data help the authors focus on developing concepts about the data, and the analyses influencing further data collection [32, 33]. Memos, reflections on the research process, and ideas for an initial analysis, were used actively throughout the process. According to the principles of grounded theory, an iterative process of data collection, coding, and analysis was employed. Participants’ main concern was identified, and their ‘solution’ to the main concern is the content of the core category [32]. In constructivist grounded theory it is acknowledged that what one discovers in data is part of one’s own perspective [32]. This version of grounded theory differs from the original grounded theory, which emphasized that the researcher should have an objective approach to data [34, 35]. According to Charmaz, researchers are part of what is studied, and theories are constructed through researchers’ involvement and interaction with the participants, as well as research practice. The constructivist approach offers an interpretation, not an exact picture, of what is studied [32].

The data collection method should be based on the research question, as should access to data [32]. For this study individual semi-structured interviews were considered appropriate for gathering data. The data in this study is based on the interviews with parents and siblings from the sub-studies by N.N. et al. [30, 31]. The data were reanalysed with the main research question: How does having a daughter/sister with an eating disorder affect the family? (The complete interview guide is included under *data collection*).

### Co-researchers

Two co-researchers (N.N,N.N) were involved as consultants in the project: one with experience of having had an eating disorder, the other with experience of being the mother of a daughter with an eating disorder. The co-researchers were involved in developing the research questions and the interview guide and participated in discussing and planning the project as a whole. N.N participated in the final analysis of the results. Their ability to view the results from a different angle, based on their experience, was a valuable contribution throughout the research process.

### Participants

Participants were parents, and siblings, of women (over the age of 18) with anorexia or bulimia. The onset of the illness was for the majority in their early teens; for a few, it was their early twenties. The daughters/sisters were between 20 and 32 years, and their mean age was 24.71. All the daughters/sisters had long duration (± 10 years) of illness and all the participants described their daughter/sister as fluctuating between better and worse periods during their eating disorder. Four of the daughters/sisters were perceived as now having control over their eating disorder and half of the group were currently under treatment. Twenty-one individual interviews were conducted, with parents and siblings from eleven families. The participants were asked about age, living situation, family, and marital status. Parents in six of the eleven families were married couples, while the remaining were divorced. Participants were recruited by one inpatient and three outpatient eating disorders and general psychiatric units, and two organisations providing support to patients and families regarding eating disorders. The units and organisations conveyed information about the project to prospective participants, who voluntarily signed up to participate. Participants were recruited from different counties within Norway. Table 1 presents characteristics of the participants.

**Table 1:** Characteristics of participants

Families	Parents Age 52–70	Siblings Age 20–31	Daughter/sister Age 20–32 Diagnosis	Current living situation of daughter/sister
Family 1	Mother Father	Sister (I) Sister (II) Brother	BN	Not living in family home
Family 2	Mother Father	Sister	AN	Partly living in family home
Family 3	Mother Father	Sister	AN	Not living in family home
Family 4	Mother	Sister	BN	Partly living in family home
Family 5	Mother Father		AN	Not living in family home
Family 6	Mother		AN	Living in family home
Family 7	Mother		AN	Not living in family home
Family 8		Sister	AN	Not living in family home
Family 9		Sister	AN	Living in family home
Family 10		Brother	AN	Not living in family home
Family 11		Brother	BN	Not living in family home

*AN* anorexia nervosa, *BN* bulimia nervosa

### Data collection

Semi-structured interviews were conducted by asking a few interview questions so that participants had the opportunity to steer the direction of the interview [32]. An interview guide was used, with a few predetermined open-ended questions. The questions are presented in Table 2.

**Table 2:** Interview guide

• What kind of experiences do you have as part of the family of a daughter/sister with an eating disorder?
• Who comprises your family, and what is the ED diagnosis of your daughter/sister?
• How does it affect/have affected your family living close to a family member with this illness?
• Are there/have there been any main challenges in your family?
• Are you using/have used certain kinds of strategies for handling the situation as a family of a daughter/sister with an eating disorder?
• Do you have experience with health services in this context? In that case, what experiences do you have?

*ED* eating disorder

The guide was adjusted as new themes were brought up [32]. By following up on codes from the early interviews, more pointed questions were developed [36]. One example is the theme of changed family dynamics, which was brought up by both parents and siblings in the first interview. They talked much about the family becoming changed as a result of everyone’s attention being placed on the family member with the eating disorder. In further interviews questions regarding new family patterns and roles were then asked. As the findings evolved, further interviews were conducted to refine the categories [32, 36], hence more participants were recruited from the recruiting units and organisations.

Thirteen interviews were conducted face-to-face and the remainder by telephone (due to Covid-19). The participants preferred telephone interviews to the option of video conferencing interviews. All interviews were audio recorded and transcribed verbatim. They each lasted between 25 and 150 min, with a majority lasting about one hour. Interviews and transcriptions were conducted by the first author. The quotes from the participants, used in this article, were translated from Norwegian.

### Data analysis

The data were analysed following the principles of constructivist grounded theory [32]. First, the transcriptions were initially coded, sticking closely to the data by studying the words, lines, and segments. In vivo codes, participants’ own terms for words or sentences, were used where appropriate. The next stage of the analysis was focused coding. Here the initial codes were studied and compared. The focus was on what the initial codes said and the comparison between them. The focused codes were constructed based on the frequency of the initial codes or their significance for the aim of the study. Decisions needed to be made about which initial codes made the most analytic sense to clearly categorize the data. This phase more clearly represents the voice of the researcher, as the focused codes are based not only on the participants’ statements but on relevance to the topic. The focused codes were then compared and merged into preliminary categories, and these categories consisted of groups of focused codes with similar characteristics [32].

Several memos were written during the process of analysis and the development of the categories. One example is a memo regarding parents’ and siblings’ expressions of feelings of responsibility for the family member with the eating disorder: *Many of the siblings also expressed that they felt a great deal of responsibility for taking care of the sister with the illness. Did this imply that the parents and the siblings both perceived the same level of responsibility?* It remained however unclear what the emerging categories were explaining, as they needed to be elaborated and refined. Hence more interviews were conducted. After further data collection, a main category with three related subcategories was identified. The last interviews did not add any particularly new information to the categories, other than some additional details. The, mostly, long interviews yielded rich data, which provided in-depth insights into the phenomenon being explored.

First author conducted the coding process. After constant comparison between data, codes, and preliminary categories, a core category was developed. To ensure a consistent process, the development of the core category was conducted in collaboration between the authors N.N,N.N,N.N,N.N.

### Ethical considerations

Participants gave informed written consent to participate voluntarily in the project and had the opportunity to withdraw at any time. The consent for participation had to first be given by the daughter/sister affected by an eating disorder, as requested by the ethical committee. Participants’ confidentiality was protected by anonymizing the data. Information about their identities was stored securely in accordance with data legislation and university procedures.

## Results

### Balancing a pattern of care within a closed family system

All the participants talked about the family member’s eating disorder as a process, from the evolvement of the illness to a still ongoing situation. Though the majority no longer lived together with the family member with the illness, their daily life was still influenced by the daughter/sister. *The family being left to themselves* was identified as the participants’ main concern. When the daughter/sister developed an eating disorder the family tried to handle the challenges related to the illness themselves, because of the lack of professional help. The families experienced being left to themselves in this situation: *They (the health services) were so dismissive, I got the impression that it was not worth spending money on eating disorders… It was not a priority. We were left astray (Mother fam. 7).* By adapting to the family member with the illness the family’s social life was also narrowed. Due to generally limited knowledge and understanding of eating disorders, the families felt insecure talking to others outside the family.

To manage the main concern the family had to *balance a pattern of care within the closed family system*; this comprises the core category of the study. The families found themselves in a demanding situation balancing several caregiving tasks: *I do not think the health services have any idea how much work it is, they put a huge responsibility on the parents. Nutrition, activities to be followed, logged, talked about… it is a full-time job (Mother fam. 7).* The family system that was being established took care of the family members with the illness as well as made it bearable for the rest of the family members: *It is difficult in relation to the ill one, and in relation to her siblings, it can be challenging in relation to your partner…(Father fam. 1).* The degree of facilitating the illness, which could imply accommodating the eating disorder, was also perceived as a balancing act: *Maybe we use too much energy in facilitating and we do not get any response from her (the daughter). So there is a little balancing act as I perceive it (Father fam. 2).*

**Fig. 1:**
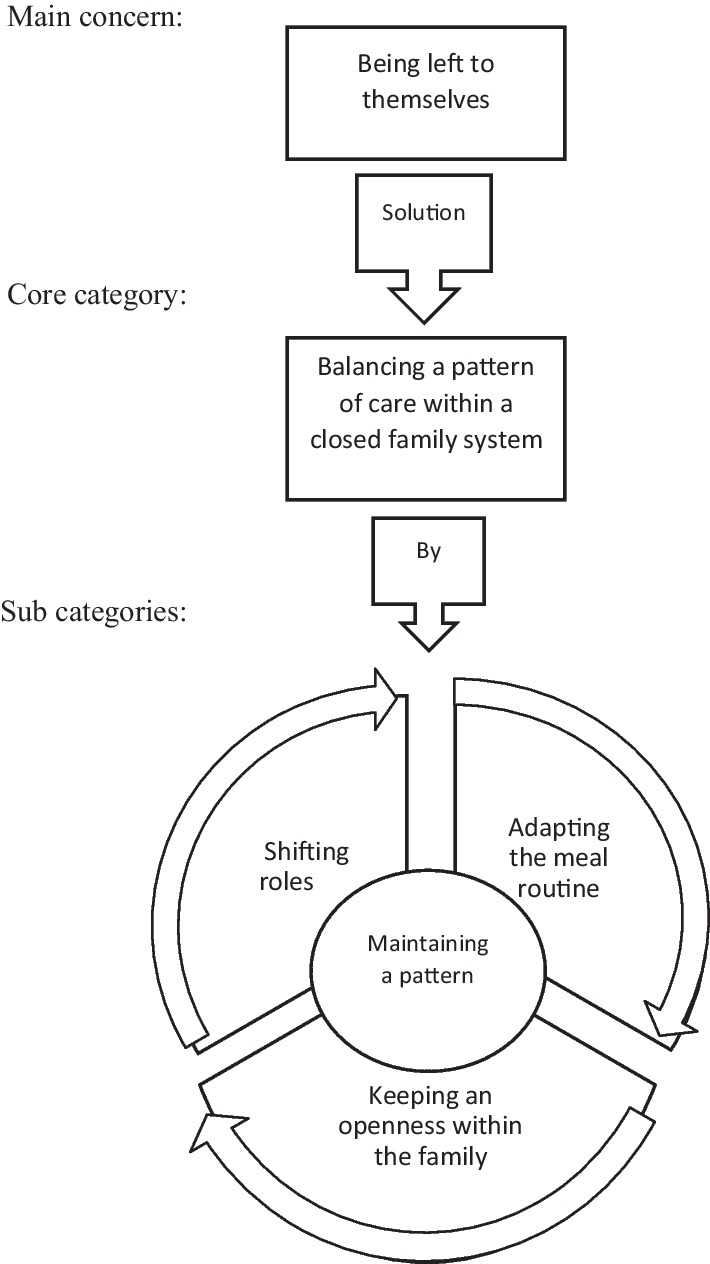
Main concern and core category with subcategories from the analysis

The family balanced a pattern of care by *shifting roles*, *adapting the meal routine,* and *maintaining an openness within the family.* Roles became shifted as both the sibling and the parents adapted to the family member with the illness. The siblings took on responsibility far beyond being a sibling, while the parents took on extensive caregiving tasks, like professional health carers, to help their ill daughters. Considerable time was spent on facilitating meals trying to meet the demands and needs of the family member with the eating disorder. Maintaining an openness within the family was elementary for making it possible to function within the closed family system. Figure 1 presents main concern and core category with sub-categories from the analysis.

### Shifting roles

The dynamic in the family changed after the daughter/sister became ill, and both the parents’ and siblings’ roles were shifted: *When someone in the family is ill, the whole dynamic in the family also becomes a little sick (Sister fam. 8)*. The siblings often experienced a greater sense of responsibility, and their role as son or daughter was downplayed. The changing family dynamics led to feelings of a lack of space for the siblings in the family: *It was not that we did not care, but we had our own lives as adolescents, but there was no room for it. The dynamics of the family changed, it became me and my younger sister, we had each other, while mom and dad were very involved with her (the ill sister) (Sister fam. 3).* Several of the siblings assumed the role of carer for their sister with eating disorder and some wanted to offload the parents’ burden: *I was really trying to be there at first because I felt that my mom and dad needed a little help (Sister I fam. 1).*

The daughter with the eating disorder often required the parents’ full attention. The parents assumed the role of professional health carers by caring for the daughter by monitoring her illness. This quote from one of the fathers reflects what the majority of parents said about their capacity to care for other siblings and their feelings of guilt concerning this: *There are siblings, two sisters, and much is prioritized in follow-up on the ill one. It is kind of unfair, I have not been there for them enough (Father fam. 3).*

### Adapting the meal routine

Adapting meals comprised a significant part of the families’ daily life. Several of the parents tried adapting the meals so that the daughter would eat something. Thus there often unfolded a constant struggle that could entail several conflicts: *All the time these negotiations when it comes to food (Mother fam 6).* The siblings also became involved in the food routine that was adapted to their sister with the eating disorder. Some experienced this food routine as having an actual impact on the rest of the family’s eating habits: *We have always eaten what we wanted, but suddenly there was such a focus on food, generally, the topic of food was consistent in every dinner at home. We all got more careful about what we actually ate, which we had never been before (Sister fam. 3).* Being a witness to bulimic behaviour during family meals was found especially challenging: *We eat together, and you see that she always leaves the table first and you know that she is going to vomit, and often you hear it… (Sister II fam. 1).*

As time went by the families perceived facilitating around meals as exhausting, but some had learned to live with the situation and had accepted it: *I just let it go, I did not even bother to be annoyed about it, just thought, ok; we all just have to eat vegetables and chicken then. … just have to put up with it (Sister fam. 2).* The families did the best they could to ensure a certain food intake for the family member with the illness. At the same time, they did not get much confirmation concerning whether what they did was right or wrong. The parents were especially often left to uncertainty concerning whether they should or could have pushed their daughter more when it came to varieties of food and intake: *Our concern was, how much can we push her, can we require something of her? (Mother fam. 5).*

### Maintaining openness within the family

Even though the family became isolated, openness within the family was considered a strength that made it bearable for the participants to maintain the pattern of care: *We are a strong family, we are very attached to each other and talk about both the unpleasant and pleasant things, so the openness in our home has been a strength of ours (Mother fam. 2).* Closer cohesion and better communication were considered positive outcomes in handling the eating disorder. Some of the family members were perceived as becoming more caring and understanding during this process, as this sister experienced her father: *I notice that he has become much more gentle. And much more responsive to feelings, that maybe did not get that much space in our upbringing (Sister fam. 8).* Some of the families had participated in multi-family therapy, and they acknowledged that this intervention helped the family to communicate better and more openly. By acquiring information about the illness and advice on how to handle it, the family members felt they received a common understanding and approach to the illness. Having open communication within the family was a work in progress for several of the families: *That is what we work on the most, somehow having open communication between all of us (Sister fam. 4).*

While multi-family therapy, support groups for eating disorders, and a few other interventions helped openness within the family, the family remained a closed system. Due to a lack of or inaccessible help for the family, as well as for the individual with the eating disorder, the caring pattern was maintained within the family. Some of the siblings eventually left their family home because they needed to distance themselves from the illness and the caring pattern; the remaining family members then maintained the pattern. The majority of the participants would have wanted more general information about the eating disorder from the health services, family therapy, or conversations that included the whole family, as well as individual support for themselves. The families’ feeling of being left alone in their situation, despite several attempts to get help, is reflected by a quote from this mother: *My experience over the years is that we stood very much alone, the feeling of loneliness in everything concerning having a daughter with that illness, there was very little understanding from others (Mother fam. 3).*

## Discussion

Our findings suggest that the families of individuals with eating disorders experienced that their family became a closed system, as they had to handle the challenges related to the illness themselves because of a lack of professional help from outside. The families had to balance a pattern of care: the parents and the siblings shifting roles regarding responsibility and care, adapting the meal routine, and maintaining openness within the family by having open communication.

The parents in the current study used most of their resources on the daughter with the eating disorder, and as reported in previous research, parents had difficulties in responding to the needs of other family members [12–14, 18–20]. Several of the siblings in the study assumed new roles entailing more responsibility in the family, and they perceived themselves positioned at the same level as the parents within the family. A study on siblings’ of adult sisters with anorexia revealed that siblings felt a strong sense of loyalty to their parents, who they perceived as being heavily burdened by the illness, while also wanting to protect their affected sibling from family conflict, perceiving themselves as mediators in the family [21]. When a family member becomes severely ill, the whole family often needs to restructure [37]. In an attempt to achieve balance and peace within the family the rest of the family members often forsake one’s own needs and feelings [37].

Families impacted by eating disorders have been observed to have more cohesion, a lower family hierarchy, more constraining family rules, and less conflict compared with control families [11]. The families in the current study appeared to have features of enmeshment, as defined by Minuchin et al., where sub-system boundaries are poorly differentiated [1]. For example, children can become elevated in the hierarchy to join the parent sub-system [1], which appeared as a pattern in the families in the current study. The boundaries that keep family members overinvolved with each other and separated from the world are typically well-defined and strong, while the boundaries within the family often are diffuse [1]. For the participating families, the dynamics changed as a result of the family having to restructure to handle the eating disorder. The family members did not describe their family patterns as previously being characterised by enmeshment, in contrast to Minuchin who was referring to a long-standing pattern that was seen as part of the transactional characteristics that were a prerequisite for the eating disorder [1].

The family’s lack of identified professional support is very likely to play into these dynamics more than pre-existing dysfunction. Because of the lack of professional support the families were left to themselves to handle the challenges related to the eating disorder. Whitney & Eisler [7] reported similar findings on how the families reorganise during the early stages of the illness. Often families know little about the illness itself and how to access the appropriate help. The families made concerted efforts to try to understand the illness and support the ill family member. This required much, especially of the parents’ resources, and siblings found themselves sidelined and did not receive the attention needed [7].

To balance a pattern of care within the family adapting meals became a central part of the families’ daily life. Previous research has reported that interactions around food and meals increasingly dominate the relationships between family members. Parents of individuals with eating disorders expressed particular concern for siblings and other family members, who witnessed the confrontation and struggles around mealtimes [7]. The siblings from the Dimitropoulos et al. study experienced a lack of normalcy in their relationship with their affected sibling and their family, especially surrounding meals, and they perceived that the parents accommodated meals and food to the sibling with the illness [21]. This is similar to the findings in the current study, and though some of these siblings even adapted the new eating routine themselves, they resigned to keep a calmer atmosphere within the family. A study on accommodation of symptoms in anorexia nervosa found that the carers ended up in a cycle, vacillating between accommodation and confrontation. For those caring for an individual with an eating disorder over a longer duration, attempting to adopt a decreased focus on food and a less confrontational approach were reported [28]. Helping families detach from behaviours that enable or accommodate the illness is an important part of treatment for eating disorders across the age and diagnostic spectrum [9]. Whether the accommodations in the current study led to maintenance of the illness remains an unanswered question. These families described being in a cycle and maintained the accommodated pattern to cope with the demanding situation.

When a family member suffers from an eating disorder the illness often becomes the central organising principle that changes the daily routines [38]. How the family handles these challenges depends on its resilience and resources [38], as well as the right care and support [15]. The participants in the current study considered open communication within the family a coping strategy in handling daily life, and this was seen as a positive outcome of the eating disorder. The families that had participated in multi-family therapy and support groups said it helped the family to better communicate and gain a common understanding and approach to the illness. However, due to a lack of continued support interventions, this openness was mainly kept within the family. Multifamily therapy for young adults with eating disorders, including parents and siblings, has generally reported positive experiences of connecting to others in similar situations and improving family members’ understanding of the illness, helping them to understand each other [39–41]. Support groups have also been reported to be highly valued by carers for providing understanding and exchanging experiences [14]. Practical information adapted to the carers’ needs, from healthcare professionals, and family-based treatment and training in how to relate to and deal with the illness, are reported interventions that led to moderate reduction in emotional stress and burden for the caregivers. It is considered good practice to work jointly with the carers and provide them with appropriate information regardless of the patient’s age [8]. A variety of psychoeducational interventions can improve carer coping and reduce distress, burden, and expressed emotions, and these changes are sustainable over time [42]. To make such changes sustainable over time, one could imagine that consistent interventions, as requested by the participating families, are crucial.

However, research on families of individuals with eating disorders have generally reported a lack of understanding and limited available formal support, which increased feelings of social isolation and frustration [14]. The openness further strengthened the unity within the participating families and thus their capability to care for the family member with the eating disorder, which in turn led to maintenance of the pattern of care.

### Strengths and limitations

A potential limitation of the study is that some of the participants were from the same families, while others were single participants from different families. One could imagine that participants from the same family would share some main viewpoints, or even feel restricted in terms of sharing their views during the interviews. Nevertheless, a high degree of consistency was found in the sibling group, as well as among the parents, regardless of which family to whom they belonged. A second limitation could be that the consent for participation had to be given by the family member affected by the eating disorder, so it is possible that these participants do not represent families affected by major conflicts or trauma. Third, the group of individuals with eating disorders mainly comprised individuals with anorexia, and this could have limited the diversity in the material.

By interviewing both parents and siblings we were able to present a family view, as previous research has recommended [11, 14]. The study’s inductive and open approach encouraged the participants to emphasize what was important to them [32]. The co-researchers’ involvement throughout the research process, with their ability to view the results from a different angle based on their own experiences, was considered to strengthen the credibility of the study [43, 44].

### Implication

The findings implicate that even though the family member affected by an eating disorder is an adult, the family often carries a great caregiving burden, which they need to offload. The health services should be aware that the families perceive the help and support provided as inaccessible and inadequate, for both the family member with the eating disorder as well as for the rest of the family. Intervention should also be adapted individually to the family members, while still protecting the integrity of the adult individual with the illness. Clarification of roles and responsibilities for the families in relation to the health services in caring for the individual with eating disorders is needed.

Previous research indicates that interventions such as multi-family therapy for adults have positive outcomes for both the individual with an eating disorder and the family [39–41]. It has been argued that irrespective of the stage of the illness, the impact on the caregivers should determine the type of interventions needed [19, 24, 45]. Future research should look deeper into how to support the families of adults with eating disorders to disrupt the pattern of becoming left to themselves, and also whether these patterns could be maintaining the eating disorder. More knowledge about the challenges of taking a family perspective concerning adults with eating disorders is needed.

## Conclusions

This study contributes to understanding how family life is affected by having an adult family member with an eating disorder. From the participating families’ perspectives, the families were being left to themselves caring for the family member with the illness due to the lack of professional help. The families became closed systems having to balance a pattern of care within the family. Roles became shifted as the participants took on extensive responsibility and caregiving tasks. Adapting the meal routine became a major part of the families’ daily life. An openness was kept within the family which made it possible to function within the closed family system. The results emphasize that the families need to offload the caregiving burden, and that help from the health services must be accessible. Support interventions should also be individually adapted to the family members.

## Data Availability

The data that support the findings of this study are available on reasonable request from the corresponding author. The data are not publicly available due to their containing information that could compromise the privacy of research participants.

## Abbreviations

AN: Anorexia Nervosa
BN: Bulimia Nervosa
ED: Eating Disorder
